# Hematopoietic Stem/Progenitor Cell Proliferation and Differentiation Is Differentially Regulated by High-Density and Low-Density Lipoproteins in Mice

**DOI:** 10.1371/journal.pone.0047286

**Published:** 2012-11-07

**Authors:** Yingmei Feng, Sarah Schouteden, Rachel Geenens, Vik Van Duppen, Paul Herijgers, Paul Holvoet, Paul P. Van Veldhoven, Catherine M. Verfaillie

**Affiliations:** 1 Interdepartementaal Stem Cell Institute, Katholieke Universiteit Leuven, Belgium; 2 Division of Cardiac Surgery, Katholieke Universiteit Leuven, Leuven, Belgium; 3 Atherosclerosis and Metabolism Unit, Department of Cardiovascular Diseases, Katholieke Universiteit Leuven, Leuven, Belgium; 4 Laboratory of Lipid Biochemistry and Protein Interactions, Department Molecular Cell Biology, Katholieke Universiteit Leuven, Leuven, Belgium; University of Padova, Medical School, Italy

## Abstract

**Rationale:**

Hematopoietic stem/progenitor cells (HSPC) are responsible for maintaining the blood system as a result of their self-renewal and multilineage differentiation capacity. Recently, studies have suggested that HDL cholesterol may inhibit and impaired cholesterol efflux may increase HSPC proliferation and differentiation.

**Objectives:**

We hypothesized that LDL may enhance HSPC proliferation and differentiation while HDL might have the opposing effect which might influence the size of the pool of inflammatory cells.

**Methods and Results:**

HSPC number and function were studied in hypercholesterolemic LDL receptor knockout (LDLr^−/−^) mice on high fat diet. Hypercholesterolemia was associated with increased frequency of HSPC, monocytes and granulocytes in the peripheral blood (PB). In addition, an increased proportion of BM HSPC was in G_2_M of the cell cycle, and the percentage of HSPC and granulocyte-macrophage progenitors (GMP) increased in BM of LDLr^−/−^ mice. When BM Lin-Sca-1+cKit+ (i.e. “LSK”) cells were cultured in the presence of LDL *in vitro* we also found enhanced differentiation towards monocytes and granulocytes. Furthermore, LDL promoted lineage negative (Lin−) cells motility. The modulation by LDL on HSPC differentiation into granulocytes and motility was inhibited by inhibiting ERK phosphorylation. By contrast, when mice were infused with human apoA-I (the major apolipoprotein of HDL) or reconstituted HDL (rHDL), the frequency and proliferation of HSPC was reduced in BM *in vivo*. HDL also reversed the LDL-induced monocyte and granulocyte differentiation *in vitro*.

**Conclusion:**

Our data suggest that LDL and HDL have opposing effects on HSPC proliferation and differentiation. It will be of interest to determine if breakdown of HSPC homeostasis by hypercholesterolemia contributes to inflammation and atherosclerosis progression.

## Introduction

Lipoproteins play essential roles in coronary heart disease (CHD). A meta-analysis showed that a 1 mmol/l reduction in the level of LDL cholesterol is associated with 19% reduction in CHD mortality [1]. In contrast, increase of HDL cholesterol by 1 mg/dl decreases the incidence of CHD 2–3% [2]. Apart from CHD, hypercholesterolemia also accelerates other diseases including Alzheimer's amyloid pathology [3].

Atherosclerosis, the underlying pathogenesis of CHD, is characterized by chronic inflammation due to both loss of endothelial integrity and to subendothelial retention of LDL [4]. Once retained in the subendothelial space, both native and modified LDL activate vascular cells, attracting circulating white blood cells to form plaques.

It is well known that granulocytes and monocytes play critical roles in atherosclerosis development. Granulocytes are the first cells recruited to the plaque, where they secrete elastase and metalloproteinase, resulting in extracellular matrix degradation and inflammatory cell adhesion [5]. Monocytes in the plaque take up both native and modified LDL and become foam cells, the hallmark of atherosclerosis [6]. Inflammatory and/or activated vascular cells also secrete inflammatory cytokines such as tumor necrosis factor (TNF)-α, interferon (IFN)-γ, granulocyte macrophage colony-stimulating factor (GM-CSF) and monocyte CSF (M-CSF), which further reinforce the inflammation processes [7], [8], [9], [10], [11], [12], [13]. Thus, hypercholesterolemia-associated leukocytosis and transformation of monocytes to macrophages facilitate atherosclerosis progression [14].

In contrast, there is an inverse correlation between the incidence of CHD and plasma levels of HDL and its major apolipoprotein A-I (apoA-I) [15]. Aside from HDL-mediated reverse cholesterol transport and anti-oxidative properties, one of the major functions of HDL is to inhibit vascular cell activation, thereby, prohibiting inflammatory cell infiltration and atherosclerotic progression [16], [17], [18]. We previously demonstrated that HDL improved endothelial repair, inhibited inflammation and thus attenuated transplant arteriosclerosis and atherosclerosis in vein grafts in the presence of Scavenger Receptor Type B-I (SR-BI) [19], [20], [21].

Recently, Yvan-Charvet *et al.*, reported the suppressive role of HDL on hematopoietic stem cell (HSC) proliferation via ATP-binding cassette transporters (ABCA1) [22]. In contrast, hypercholesterolemia has been shown to promote BM cell mobilization in man and mice [23], [24]. These studies lead to the hypothesis that HDL and LDL may have opposing effects on hematopoietic cell function. We demonstrate here that hypercholesterolemia is associated with enhanced proliferation of HSPC *in vivo* and with increased myeloid cell differentiation. Both appear to be mediated, at least in part, by extracellular signal regulated kinase (ERK). By contrast, HSPC proliferation was inhibited in BM of C57BL/6J mice infused with purified human apoA-I or reconstituted (r)HDL. We further demonstrated that exposure of HSPC to LDL *in vitro* induced differentiation to monocytes and granulocytes, whereas HDL decreased myeloid cell differentiation induced by LDL.

## Materials and Methods

### Mice

Wild type C57BL/6J (CD45.2) and B.6SJL-PTPRCA (CD45.1) mice, maintained in the animal facility of the Katholieke Universiteit Leuven, were used at the age of 2–3 months. rHDL and human apoA-I infusion experiments were performed in C57BL/6 mice. In brief, male C57BL/6J mice received saline, PLPC (1-palmitoyl-2-linoleoyl-*sn*-glycerol-3-phosphocholine) (8 mg/kg, Avanti Polar Lipids, Alabaster, Alabama, USA), human apoA-I (8 mg/kg) or rHDL (4–16 mg/kg) on days 1, 3 and 5, via tail vein. Purified human apoA-I and rHDL were kindly provided by Professor Kerry-Ann Rye (The Lipid Research, Heart Research Institute, Sydney, Australia). Homozygous LDL receptor knockout (LDLr^−/−^) mice were purchased from Jackson Laboratory (Bar Harbor, Maine). They were backcrossed with C57BL6J mice for at least 10 generations to achieve 99.9% C57BL6J background. At 8 weeks of age, LDLr^−/−^ mice were placed on high fat diet (34% fat, 1% cholesterol, Catalog no. D12492 mod, BioServices) or normal diet for 2 months. Approximately, 200 mice were used in this study. All experiments were performed with approval of the ethical committee of the Katholieke Universiteit Leuven.

### White blood cell counts

Leukocytes, lymphocytes, monocytes and granulocytes in PB were quantified with an Ac-Tdiff hematology analyzer (Beckman Coulter; Brea, CA, U.S.A.).

### ELISA for human apoA-I

Human apoA-I levels in plasma and fractions obtained by gel filtration were determined by sandwich ELISA (MyBioSource, San Diego, CA, U.S.A.).

### PLPC determination

PLPC was purchased from Avanti Polar Lipids. After reconstitution in PBS, PLPC concentration was determined using the EnzyChrom Phospholip Assay Kit (BioAssay Systems, Hayward, CA, U.S.A.).

### Separation of plasma lipoproteins by gel filtration

Mouse plasma lipoproteins were fractionated by fast performance liquid chromatography gel filtration of 50 µl plasma on Superose 12 HR 10/30 (Pharmacia, Herts, UK). Cholesterol content in non-HDL fractions and HDL fractions was quantified by Amplex™ Red Cholesterol Kit (Molecular Probe, CA, U.S.A.).

### LDL and HDL isolation by density gradient ultracentrifugation

Plasma LDL (1.019 g/ml<d<1.063 g/ml) and HDL (1.063 g/ml<d<1.21 g/ml) were isolated from healthy volunteers by density gradient ultracentrifugation in a swing-out rotor as described [25]. Subsequently, LDL and HDL were dialyzed against 1 mM EDTA in PBS overnight. Cholesterol concentration was measured by Amplex™ Red Cholesterol Kit.

### Identification of lipoprotein fractions by SDS-PAGE

To study the apolipoprotein profiles in the fractions, 20 µl of each fraction was separated on 4–12% NuPage (Invitrogen, Gent, Belgium) and then stained with GelCode Blue Stain (Thermo Fisher Scientific, Rockford, IL, U.S.A.) overnight.

### Quantification of murine apoA-I levels

Murine apoA-I expression in plasma was quantified by western blot as described before [26]. A goat anti-mouse apoA-I antibody (sc-23606, Santa Cruz Biotechnology, CA, U.S.A) was used to detect murine apoA-I, without cross-reactivity with human apoA-I.

### Lineage negative cell and LSK cell isolation

Total BM cells (TBMC) were obtained by flushing tibias and femurs. Lineage negative (Lin-) cells were isolated with the Lineage Negative Selection Kit (Stem Cell Technologies, Vancouver, Canada). Lin- cells were stained with lineage cocktail APC (Ter119/CD3e/CD11b/CD45R/B220/Ly6G/LY-6C from BD Biosciences, Franklin Lakes, NJ, U.S.A.), Sca-1 FITC (eBioscience, San Diego, CA, U.S.A.) and cKit PE (eBioscience). LSK cells were sorted on a FACS Aria III (Becton Dickinson, NJ, U.S.A.).

### Fluorescence-activated cell sorting (FACS)

Multicolor analysis for hematopoietic stem/progenitor cells (HSPC) in BM, peripheral blood (PB) or cultured cells was performed on a FACScanto (Beckton Dickinson). Surface markers for identification of HSPC, long term repopulating HSC (LT HSC) and granolucyte-macrophage progenitors (GMP) were used as previous described [27]. Briefly, HSPC were defined as Lin− Sca-1+ cKit+ cells (so called “LSK cells”); LT HSC were identified as CD34−/Flk2−/LSK cells; and GMP were defined as CD34+ FcR+ Lin− Sca-1− cKit+ cells. To quantify HSPC in the circulation, 200 µl PB was treated with Ammonium Cloride (StemCell technologies, Vancouver, Canada) to remove red blood cells before staining. CD34 FITC, Sca-1 PerCP-Cy5.5, Flk-2 PE, cKit PE, FcR PerCP-Cy5.5, integrin β1 PE and cKit-APC-Cy7 were purchased from eBioscience. Alexa Fluor 488 conjugated phospho-p44/42 MAPK (Erk1/2) and rabbit anti-mouse SR-BI were purchased from Cell Signaling Technology (Bioké, Leiden, the Netherlands). Goat anti-rabbit alexa 488 was purchased from Invitrogen. All other antibodies were obtained from Becton Dickinson Biosciences (NJ, U.S.A.).

For *in vivo* BrdU analysis of HSPCs, mice were injected with 0.2 mg BrdU/g intraperitoneally 12 h before analysis [28]. After staining with Lineage cocktail APC, Sca-1 PE and cKit-APC-Cy7, cells were permeabilized and stained with anti-BrdU FITC using the BrdU Flow Kit according to manufacturer's instruction (Becton Dickinson).

To evaluate SR-BI expression on HSPC, TBMC were stained with rabbit anti-mouse SR-BI (1 µg/1×10^6^ cells), followed by goat anti-rabbit Alexa 488 (1/400) before performing LSK staining.

To study ERK phosphorylation in HSPCs, BM cells were stimulated with LDL, fixed, permeabilized and stained with anti-phosphor-p42/44MAPK Alexa 488, Lineage cocktail APC, Sca-1 PE and cKit-APC-Cy7 according to the manufacturer's instruction (BD Biosciences).

To study adhesion molecules expression, Lin- cells were exposed to 0 or 100 µg/ml LDL for 24 hours. After harvest, cells were stained with Lineage cocktail APC, Sca-1 FITC, cKit APC-Cy7 together with CXCR4 PE, integrin β1 PE, or integrin α5 PE for FACS analysis.

All FACS studies were performed using the appropriate isotype control antibodies. To achieve reliable quantification, at least 100,000 events were acquired.

### qRT-PCR

Total RNA from cultured Lin- cells was extracted using RNAeasy microkit (Qiagen, Valencia, CA). mRNA was reverse transcribed to get cDNA using Superscript III reverse Transcriptase (Invitrogen). Primers used in this study are as following: SR-BI: forward 5′-GGCTGCTGTTTGCTGCG-3′ and reverse 5′-GCTGCTTGATGAGGGAGGG-3′; LDLr: forward 5′-AGGCTGTGGGCTCCATAGG-3′ and reverse 5′-TGCGGTCCAGGGTCATCT-3′; ABCA1: forward 5′-AGCCAGAAGGGAGTGTCAGA-3′ and reverse 5′-CATGCCATCTCGGTAAACCT-3′; ABCG1: forward 5′-TTCCCCTGGAGATGAGTGTC-3′ and reverse 5′-CAGTAGGCCACAGGGAACAT-3′; and β-actin forward 5′-CACCACACCTTCTACAATGAG-3′ and reverse 5′-GTCTCACCAATGATCTGGGTC-3′.

### HSPC *in vitro* differentiation assay

LSK cells were cultured at a density of 1000 LSK cells per well in SFEM medium supplemented with SCF (20 ng/ml), IL-3 (10 ng/ml) and IL-6 (10 ng/ml) (all from R & D Systems). Immediately after seeding, LDL (100 µg/ml) or LDL (100 µg/ml) plus HDL (600 µg/ml) were added. In parallel, GM-CSF (10 ng/ml) was used as a positive control. After 14 days, cells were harvested by cytospin and a Giemsa stain was performed. Promonocytes were identified based on an increased nuclear/cytoplasmic ratio, and granulocyes were identified based on their specific nuclear morphology [29]. Total cells, promonocytes and granulocytes were counted under the microscope to calculate the percentage of differentiated cells. For each condition, at least 5 fields of cells were counted. After 14 days, cells were harvested and stained with antibodies against Ly-6c, CD11b, Ly-6G and F4/80 for FACS. For pERK inhibitor experiments, U0126 (10 µM) (Merck, Darmstadt, Germany) was immediately added to LSK cells upon seeding and maintained till harvest.

### In vitro adhesion and migration assay

Adhesion and migration of Lin- cells were tested as described before [19], [30]. In brief, Lin- cells isolated from CD45.2 mice were cultured with LDL 0, LDL 0 plus U0126, LDL 100 µg/ml or LDL 100 µg/ml plus U0126 for 24 hours. After numeration, 50,000 cells were allowed to adhere to fibronectin (25 µg/ml)-coated 96-well plate for 24 hours. After extensive wash with PBS, adhered cells were fixed with 3.7% paraformaldehyde, stained with crystal violet (5 mg/ml in 2% ethanol, Sigma-Aldrich) and counted under the microscope.

To test motility, 50,000 cells were loaded in the upper chamber of modified Boyden chambers (8 µm pore size, Costar, Avon, France). The lower chamber was filled with IMDM medium. After 4 hours, cells in the upper chamber were removed by swaps. Cells migrating on the lower surface of the transwells were stained with Hoechst and counted under the fluorescent microscope as described before [19]. For the adhesion and migration assays, 5 random fields were counted and the average cell number was calculated.

### Statistics

Data are expressed as mean ± SEM. Unpaired, 2-tailed Student's t test was used if there were 2 experimental groups. For more than 2 experimental groups, one-way ANOVA with Dunnett was applied to test treated groups against control or one-way ANOVA with Bonferroni was used to compare all groups. Statistical analysis was performed using GraphPad Prism (GraphPad Software Inc, La Jolla, CA, U.S.A.). A P value less than 0.05 was considered significant.

## Results

### Hypercholesterolemia leads to increased leukocytosis in blood

We hypothesized that LDL may cause proliferation and mobilization of HSPC from the BM, whereas HDL would have the opposite effects. LDLr deficiency causes impaired LDL clearance, resulting in high plasma levels of LDL-cholesterol. Thus, LDLr^−/−^ mice were used to study the effect of LDL on HSPC. LDLr^−/−^ mice received either normal or high fat diet from the age of 2 months onwards and this for 2 months. In parallel, WT mice were included as control. The lipoprotein profile of these mice is shown in Table 1 (n = 5 for both WT and high fat diet; n = 11 for normal diet respectively).

**Table 1:** Plasma lipoprotein profiles and leukocyte count in WT mice and LDLr^−/−^ mice on normal and high-fat diet.

	WT mice	LDLr^−/−^ mice on ND	LDLr^−/−^ mice on HFD
Total cholesterol (mg/dl)	66±3.3	100±9.6*	591±176.9* ^,^ #
LDL cholesterol (mg/dl)	10±1.3	44±8.4*	431±161.2* ^,^ #
HDL cholesterol (mg/dl)	50±4.3	50±5.7	152±19.6#
Triglyceride (mg/dl)	61±4.6	84±10.0	242±77.6* ^,^ #
White blood cells (k/µl)	6.2±0.68	6.3±0.37	8.6±0.44* ^,^ #
Neutrophils (k/µl)	0.6±0.13	0.7±0.12	1.1±0.19#
Lymphocytes (k/µl)	5.2±0.48	5.2±0.38	6.7±0.39
Monocytes (k/µl)	0.08±0.26	0.2±0.02	0.4±0.05* ^,^ #

Cholesterol data are expressed as mg/dl and presented as means ± SEM. Peripheral white blood cell data are expressed as k/µl and presented as means ± SEM. ND indicates normal diet. HFD means high-fat diet. WT mice: n = 5; LDLr^−/−^ mice on ND: n = 12; LDLr^−/−^ mice on HFD: n = 5.

* *P*<0.05 when compared with mice on WT mice.

# *P*<0.05 when compared with mice on normal diet.

**Figure 1:**
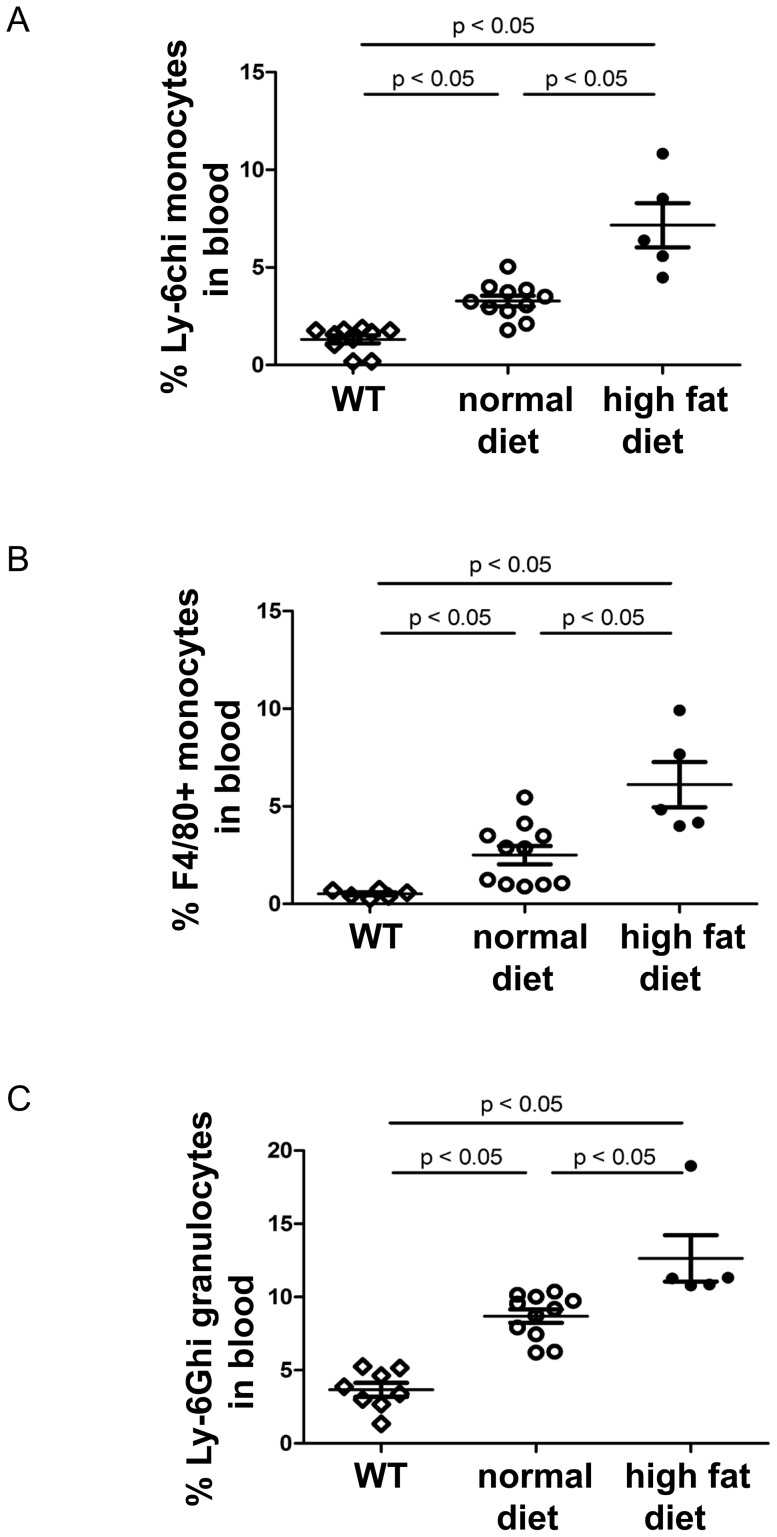
Hypercholesterolemia promotes leukocytosis in peripheral blood of LDLr^−/−^ mice. Hypercholesterolemic LDLr^−/−^ mice were used to study the effect of LDL on HSPC. At the age of 8 weeks, mice were placed either on normal diet or on high fat diet for 2 months. Two months after normal or high fat diet, blood cells of LDLr^−/−^ mice were stained with antibodies against Ly-6c, F4/80, Ly-6G and CD11b. The percentage of Ly-6c^hi^ monocytes (A), F4/80+ monocytes (B) and Ly-6G^hi^ granulocytes (C) was quantified by FACS.

Although no differences in white blood cells were observed between WT and LDLr^−/−^ mice fed a normal diet, the number of white blood cells, neutrophils and monocytes in the PB was 1.4- fold (*P*<0.05), 1.6-fold (*P*<0.05), and 2-fold (*P*<0.05) higher in LDLr^−/−^ mice maintained on high fat diet (n = 5 compared to mice on normal diet n = 11, Table 1). Furthermore, the percentage of Ly-6c^hi^ and F4/80^+^ monocytes and Ly-6G^hi^ granulocytes was increased in LDLr^−/−^ mice on normal diet, compared to WT mice. (n = 6–12, *P*<0.05) (Figure 1, A–C). Moreover, the percentage of Ly-6c^hi^ and F4/80^+^ monocytes and Ly-6G^hi^ granulocytes was 2.2-, 2.4-, and 1.5- fold higher in LDLr^−/−^ mice on high fat diet, respectively, compared to LDLr^−/−^ mice on normal diet (n = 5–12, *P*<0.05) (Figure 1, A–C and Figure S1).

### Hypercholesterolemia enhances the frequency of HSPC in the bone marrow and peripheral blood of LDLr^−/−^ mice

**Figure 2:**
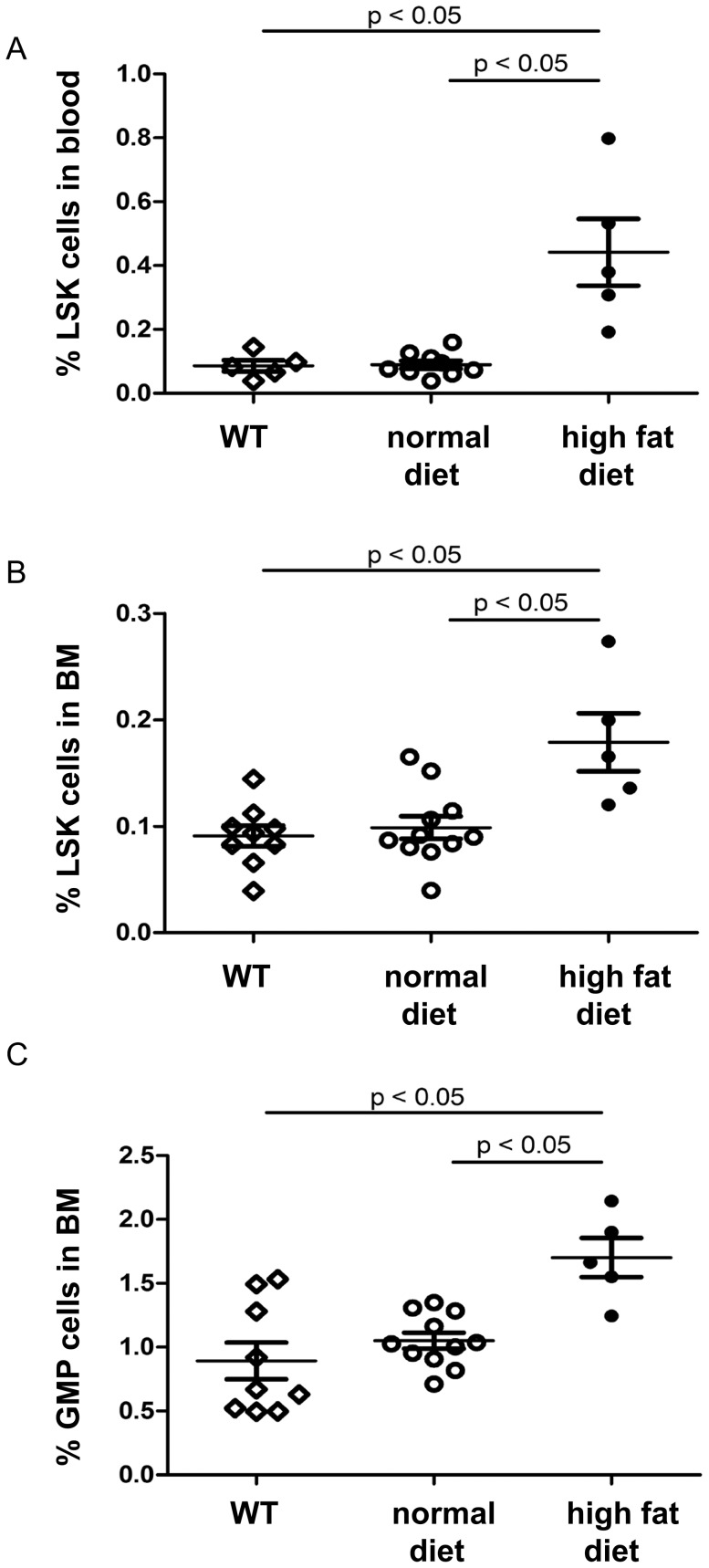
Hyercholesterolemia induced increased LSK percentage in PB and BM in LDLr^−/−^ mice. (A) Quantification of LSK cells in the PB of WT and LDLr^−/−^ mice two months after normal and high fat diet. n = 5–12. (B) The percentage of LSK cells in BM of WT and LDLr^−/−^ mice on normal and high fat diets. (C) Quantification of granulocyte/macrophage progenitors (GMP) in BM of WT and LDLr^−/−^ mice on normal and high fat diets.

Next, we measured the frequency of HSPC in PB and BM of WT and LDLr^−/−^ mice that received either normal or high fat diet for 2 months. Quantification of Lin− Sca1+ cKit+ (LSK) cells in blood showed that the percentage of HSPC in LDLr^−/−^ mice on high fat diet was increased compared to WT and LDLr^−/−^ mice on normal diet (Figure 2A). In agreement with other reports [22], [23], [31], [32], [33], [34], [35], the percentage of LSK cells in BM of WT and LDLr^−/−^ mice fed on normal diet was 0.091±0.0098% and 0.099±0.0101%, respectively (n = 9 for WT mice and n = 11 for LDLr^−/−^ mice, *P* = 0.7). However, the percentage of LSK cells in the BM was 1.9- fold higher in LDLr^−/−^ mice fed on high fat diet, compared with LDLr^−/−^ mice on normal diet (n = 5–11, *P*<0.01) (Figure 2B). We found a 1.9-fold increase of the number of CD34−/Flk2−/LSK cells in BM of LDLr^−/−^ mice on high fat diet, compared to LDLr^−/−^ mice on normal diet (normal diet: 0.018±0.0027%; high fat diet: 0.040±0.0058%, n = 5–7, *P*<0.05). Similarly, the percentage of granulocyte-macrophage progenitors (GMP) was higher in LDLr^−/−^ mice on high fat diet, compared to WT and LDLr^−/−^ mice on normal diet (LDLr^−/−^ on high fat diet: 1.7±0.04%; LDLr^−/−^ on normal diet: 1.1±0.07%; WT mice: 0.9±0.14%, n = 5–11, *P*<0.05 for both) (Figure 2C).

### rHDL infusion decreases the frequency of LSK cells in the bone marrow and peripheral blood of mice but does not affect monocyte frequency

**Figure 3:**
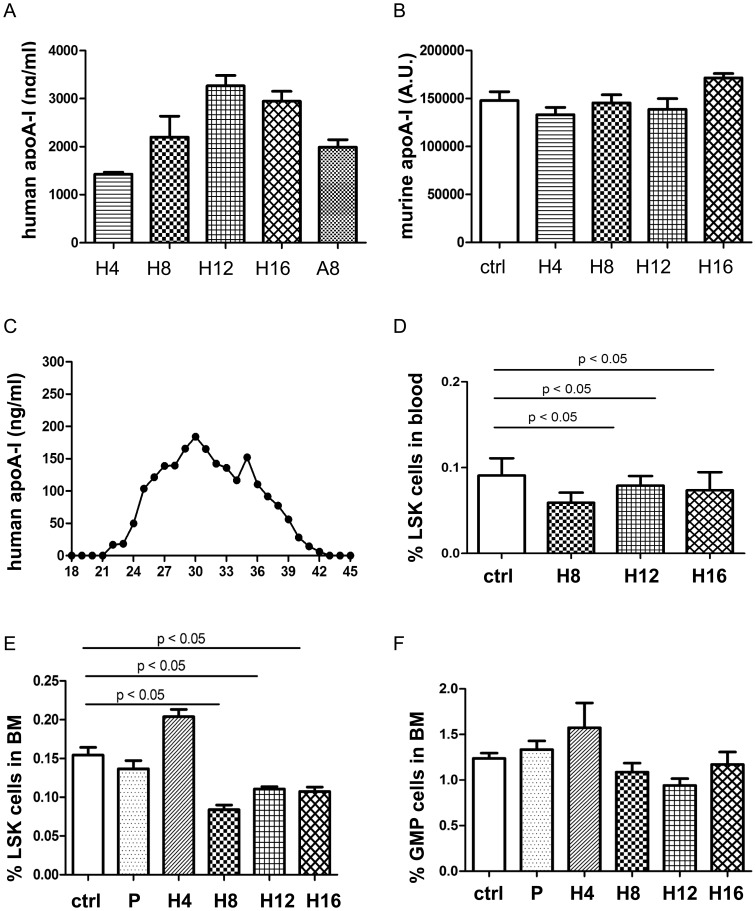
Dose response effect of rHDL infusion on HSPC in vivo. To study the effect of HDL on HSPC, C57BL/6 mice were infused with purified human apoA-I at 8 mg/kg or different concentrations of rHDL (0, 4, 8, 12, or 16 mg/kg) every 2 days for three injections. The day after the last infusion, mice were sacrificed to collect plasma. rHDL particles were identified by non-denatured gel electrophoresis (data not shown). (A) Human apoA-I levels were determined by human apoA-I EIA kit. Hx indicates rHDL infused at ×mg/kg. A8 means apoA-I infusion at 8 mg/kg. (B) Murine apoA-I levels were measured by western blot. (C) Human apoA-I levels in FPLC fractions. Lipoprotein fractions were obtained by separating plasma of rHDL (8 mg/kg)-infused mice on Sepharose 12 HR 10/30 column (flow 0.4 ml/min; 1 ml fractions, collect from 18 min on). Human apoA-I was measured in each fraction by ELISA. As a reference, fractions (30 µl per fraction) were separated in SDS-PAGE and performed GelCode Blue Stain. Cholesterol content in fractions was determined (data not shown). (D–E) LSK frequency in the PB (D) and BM (E) of C57BL/6 mice infused with saline, PLPC or rHDL (n = 5–7 for PB and n = 6–22 for BM). (F) GMP percentage in the BM of C57BL/6 mice after saline, PLPC or rHDL infusion. Ctrl indicates saline and P means PLPC.

To assess the effect of HDL on HSPC *in vivo*, C57BL/6 mice were infused with rHDL at 0, 4, 8, 12, or 16 mg/kg intravenously on days 1, 3 and 5. On day 6, LSK cells were enumerated in PB and BM. In line with other reports [18], [36], [37], [38], we showed that rHDL infusion induced human apoA-I expression without any effect on murine apoA-I expression and lipoprotein profile. The lipoprotein profile in mice infused with saline or rHDL 8 mg/kg is shown in Table 2. Both rHDL and human apoA-I infusion induced human apoA-I expression in plasma (Figure 3A). Murine apoA-I expression in plasma is shown in Figure 3B. Moreover, the injected human apoA-I facilitated HDL formation as evidenced by its presence in lipoprotein fractions obtained by FPLC (Figure 3C). Overall, no difference in total white blood cell count was seen in the blood of animals infused with the different rHDL concentrations (table 2). The percentage of LSK cells was decreased in the PB (*P*<0.05; n = 6) and BM (*P*<0.05; n = 6–22) of mice infused with 8, 12 and 16 rHDL mg/kg, respectively (Figure 3, D and E). The percentage of GMP cells in BM was not different among the three groups (Figure 3F). Representative dot plots of quantification of LSK in LDLr^−/−^ and rHDL-infused mice are shown in Figure S2.

**Table 2:** Plasma lipoprotein profiles and leukocyte count in WT mice infused with saline and rHDL 8 mg/kg.

	Saline	rHDL 8 mg/kg
Total cholesterol (mg/dl)	60±4.9	63±1.9
LDL-cholesterol (mg/dl)	12±0.8	14±1.6
HDL-cholesterol (mg/dl)	47±5.5	48±2.7
Triglyceride (mg/dl)	72±8.6	63±9.4
White blood cells (k/µl)	4.6±0.90	3.8±0.24
Neutrophils (k/µl)	1.6±0.70	0.6±0.10
Lymphocytes (k/µl)	2.6±0.25	2.9±0.21
Monocytes (k/µl)	0.3±0.11	0.2±0.04

Cholesterol data are expressed as mg/dl and presented as means ± SEM. Peripheral white blood cell data are expressed as k/µl and presented as means ± SEM. Saline group: n = 4–6; rHDL group: n = 5.

### SR-BI is expressed on HSPC and apoA-I infusion has similar effect as rHDL

**Figure 4:**
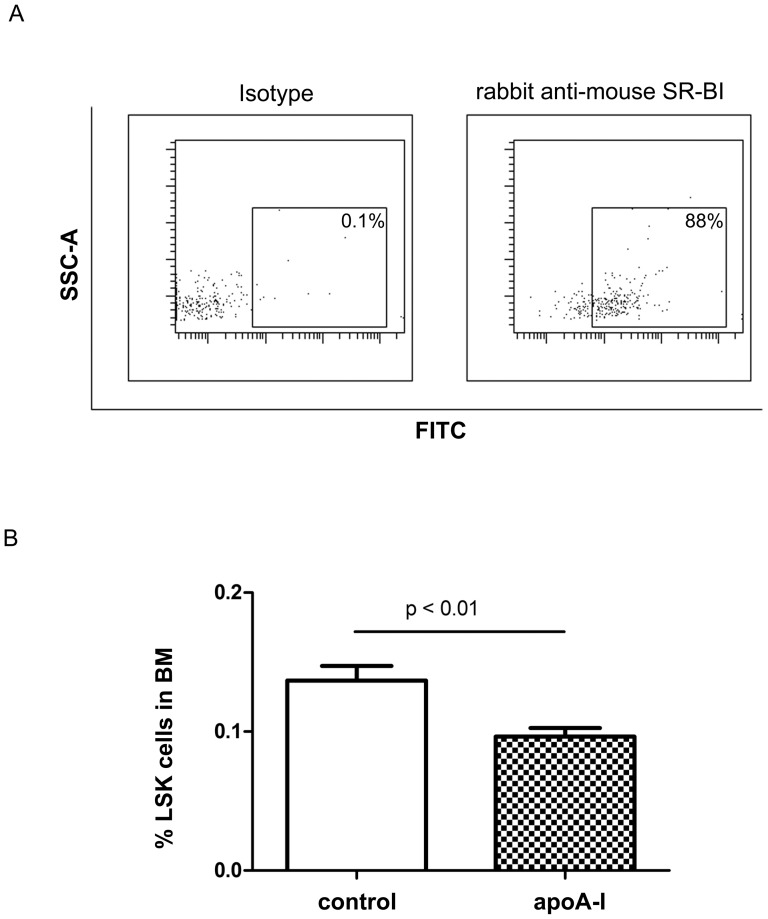
SR-BI expression on HSPC and the effect of apoA-I infusion on HSPC in PB and BM. (A) TBMCs were stained with rabbit anti-mouse SR-BI and then goat anti-rabbit Alexa 488, followed by LSK staining. SR-BI expressing LSK cells were quantified by FACS based on isotype control stains. Data are expressed as the percentage of SR-BI+ LSK cells in LSK cell population. (B) The percentage of LSK cells in BM of mice infused with saline vs. purified human apoA-I 8 mg/kg every 2 days for 3 injections.

Receptors for HDL include ABCA1, ABCG1 and SR-BI. To determine which of these receptors is expressed on HSPC, we performed in an initial step qRT-PCR to quantify the expression of *Abca1*, *Abcg1* and *Sr-BI* [39] in Lin- cells of C57BL/6 mice. This demonstrated that Lin- cells express all three receptors (data not shown). We also detected SR-B1 on the cell membrane of 88±2% of LSK cells (n = 3, Figure 4A). As apoA-I is the major apolipoprotein of HDL and HDL binds to SR-BI via apoA-I, we tested if, like rHDL, *in vivo* infusion of apoA-I would affect HSPC frequency. C57BL/6 mice were infused with saline or apoA-I at 8 mg/kg on days 1, 3 and 5, and LSK cells were quantified on day 6 by FACS. As for rHDL infusions, the percentage of LSK cells was decreased by 30% in the BM of mice that received apoA-I infusions, (n = 4–5, *P*<0.05), compared to control (Figure 4B).

### LDL and HDL have opposing regulatory effect on LSK proliferation in BM

**Figure 5:**
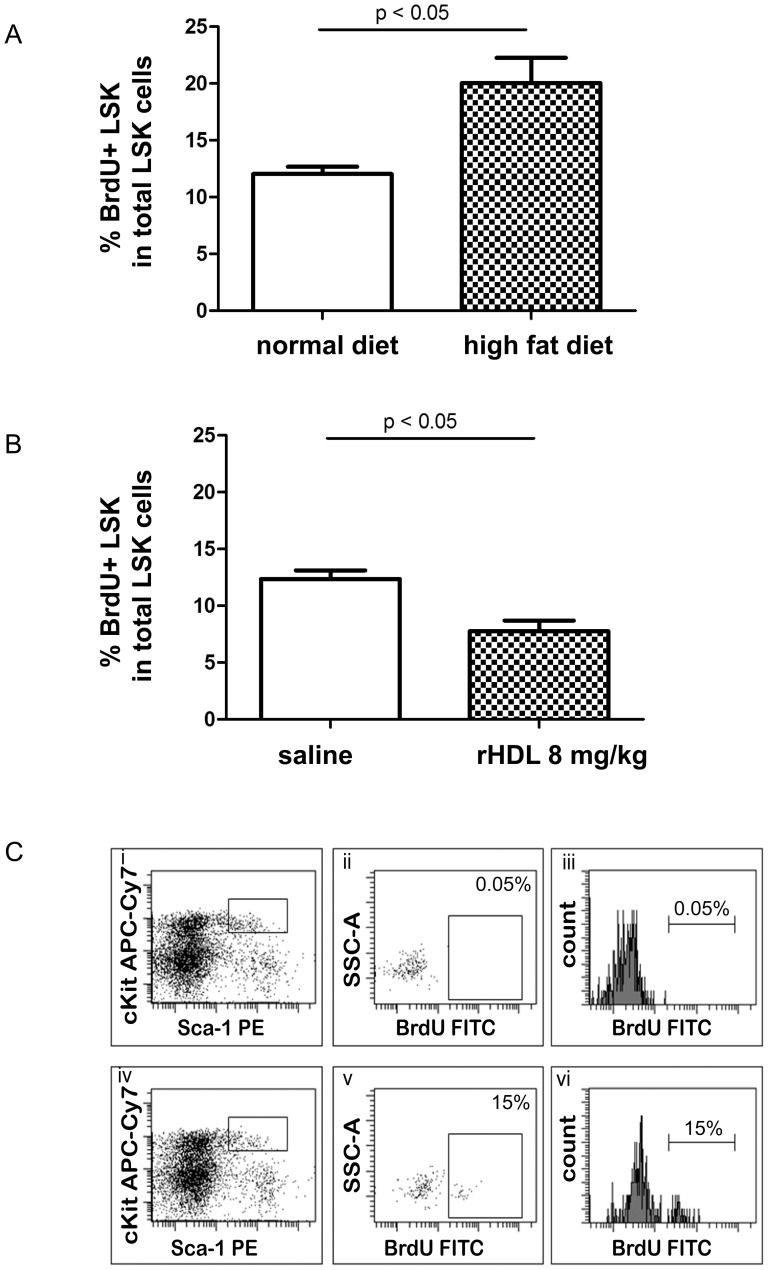
LDL promotes but HDL inhibits HSPC proliferation in vivo. BrdU was injected at 1 mg per 6 g of mouse body weight i.p. in LDLr^−/−^ mice on normal or high fat diet and the percent BrdU+ cells in total LSK cells was enumerated by FACS (A). n = 5–6. (B) Similarly, BrdU was injected to WT mice infused with rHDL 0 or 8 mg/kg 12 hours prior to dissection. The BrdU-incorporating LSK cells were quantified by FACS. Data were expressed as the percentage of BrdU+ cells in LSK population. n = 5–6. (C) Representative dot plots demonstrated BrdU-incorporating LSK cells when gated in LSK population. In the upper panel, BMC were stained with LSK but not anti-BrdU antibody: i. Cells in the box were LSK cells when gated on Lin- population; ii. Dot plot of negative control of BrdU in LSK cells; iii. Histogram of negative control of BrdU in LSK cells. In the lower panel, BMC were stained with LSK as well as anti-BrdU antibody: iv. Cells in the box were LSK cells when gated on Lin− population; v. In the Dot plot, cells in the box were BrdU-incorporating LSK cells; vi. Histogram of BrdU-incorporating LSK cells. An arrow indicates BrdU-incorporating LSK population.

To explore whether the increased LSK cell frequency is due to enhanced proliferation, we studied the proliferative status of HSPC in the BM of LDLr^−/−^ mice on normal or high fat diet. We injected Bromodeoxy-Uridine (BrdU) i.p. in LDLr^−/−^ mice on high fat vs. normal diet 12 hours prior to sacrifice, and quantified the percentage of proliferating HSPC by FACS. We found that the percentage of BrdU-incorporating LSK cells in the whole LSK cell population was increased from 12.0±0.64% in control mice to 20±2.24% in mice on high fat diet (n = 5–6, *P*<0.05) (Figure 5A).

We also tested the effect of HDL on the proliferative status of HSPC *in vivo*. C57BL/6J mice received 8 mg/kg rHDL on days 1, 3 and 5, and BrdU was injected i.p. 12 hours prior to sacrifice on day 6. The percentage of BrdU-incorporating LSK cells in the entire LSK cell population was significantly lower in rHDL-infused animals compared to controls (12.4±0.73% vs. 7.8±0.95%, *P*<0.05; n = 5) (Figure 5B). Representative BrdU incorporating LSK cells are shown in Figure 5C.

### LDL and HDL modulate HSPC differentiation in an opposite way *in vitro*

**Figure 6:**
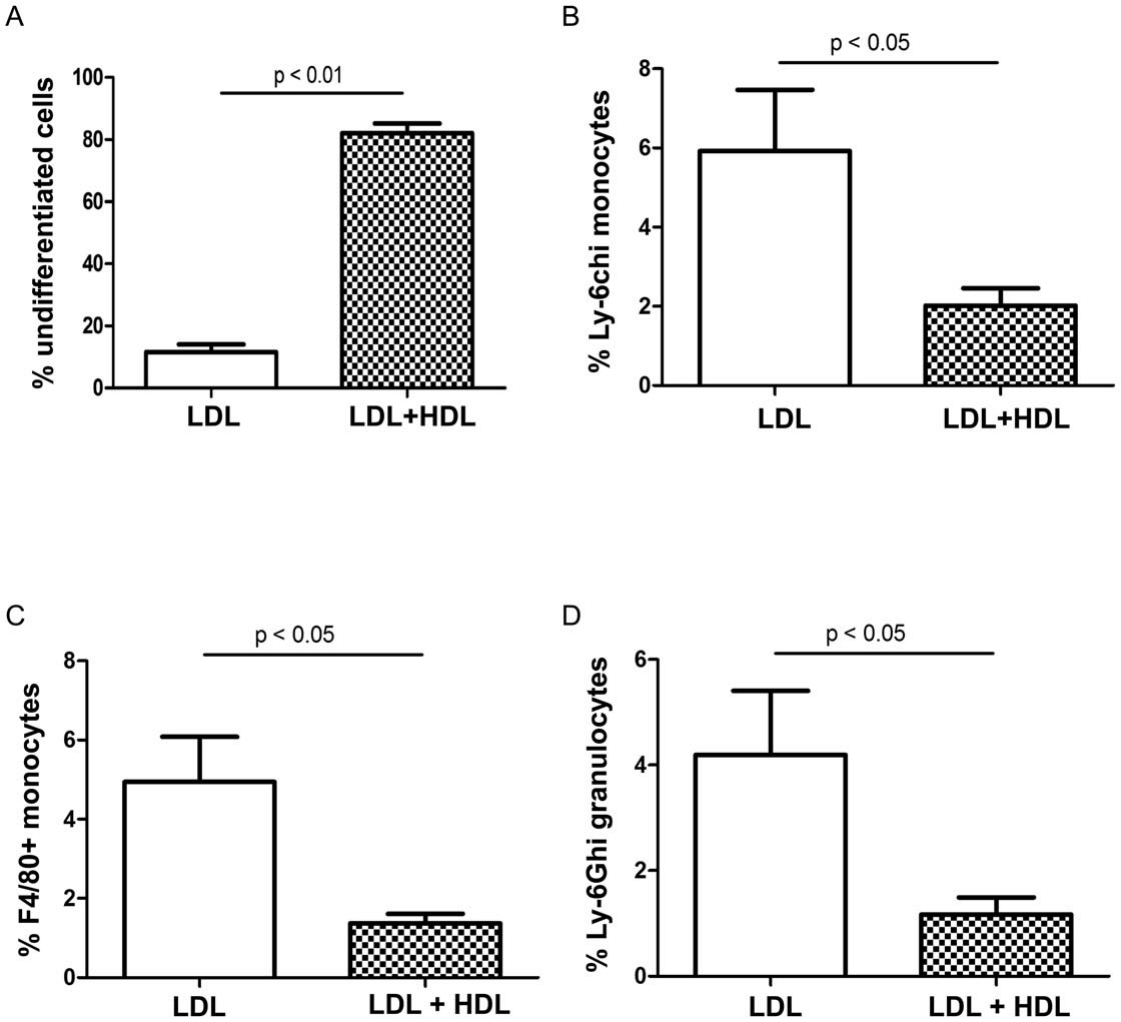
LDL increased myeloid cell production in vitro. Sorted LSK cells of C57BL/6 mice were seeded at 1000 cells per well in 96-well plate and cultured in SFEM supplemented with IL-3, IL-6 and SCF for 14 days. LDL or LDL plus HDL were added as indicated. GM-CSF was used as the positive control. Total cells and cells with morphological features of differentiated cells were enumerated under the microscopy. (A) Data were expressed as the percentage of undifferentiated cells in total cells. Cells were stained with antibodies against CD11b PE and Ly-6c PE-Cy7 (B), CD11b PE and F4/80 APC-Cy7 (C), and CD11b PE and Ly-6G APC (D) for FACS analysis.

As we had noticed an increase in monocytes and myeloid progenitor cells in LDLr^−/−^ mice on high fat diet but not in rHDL-infused animals, we next evaluated the effect of LDL and HDL on LSK cell differentiation to monocytes and granulocytes *in vitro*. LSK cells from WT C57Bl/6 mice were cultured in serum free medium supplemented with cytokines and with either LDL (100 µg/ml), or a combination of LDL plus HDL (600 µg/ml). In parallel, GM-CSF (10 ng/ml) was added as a positive control. After 14 days, cells were harvested and a Giemsa stain was performed. Addition of LDL to LSK cell cultures induced a 93±16.1-fold increase in the total cell number, whereas addition of HDL+LDL only induced a 17±7.8-fold increase (n = 4, *P*<0.01). LDL and GM-CSF induced monocytic and granulocytic differentiation of LSK cells, as assessed by immunohistochemistry [29]. However, addition of HDL decreased the LDL-induced HSPC differentiation (Figure 6A and Figure S3 representing the Giemsa stained progeny from different cultures). Cultured progeny was also stained with antibodies against CD11b, F4/80, Ly-6G and Ly-6c. Consistent with the morphological assessment, the percentage of Ly-6c^hi^ monocytes and F4/80^+^ monocytes was 8.2±0.88% and 5.4±1.73% in cells treated with LDL, but decreased to 2.6±0.49% and 1.5±0.40% in cells treated with LDL plus rHDL (*P*<0.5, for both) (Figure 6, B and C). In addition, the percentage Ly-6G+ granulocytes was 4±1.27% in LDL-treated cells but decreased to 0.9±0.25% in the presence of HDL (p<0.05) (Figure 6D).

### LDL-induced HSPC differentiation toward granulocytes is mediated via pERK

**Figure 7:**
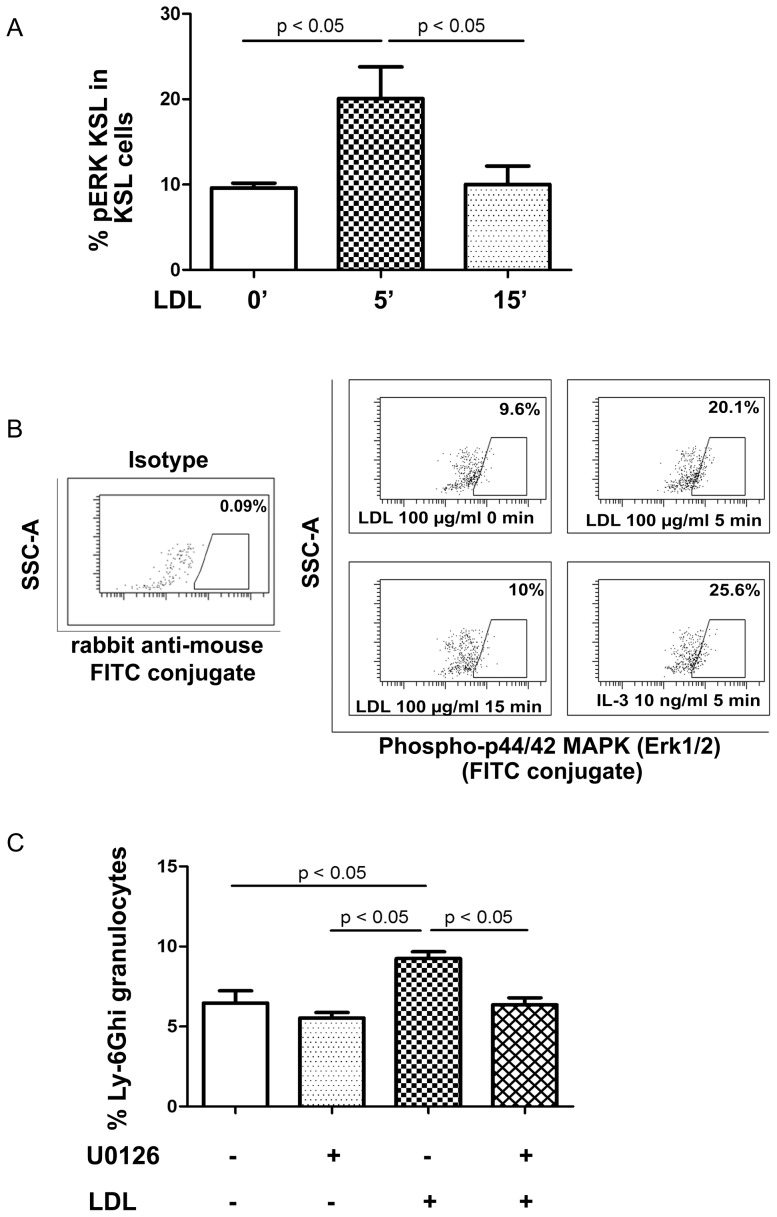
LDL modulates LSK cell differentiation toward granulocytes in an Erk1/2 dependent manner. (A) Kinetic analysis of ERK phosphorylation was done following LDL (100 µg/ml) stimulation of TBMCs for 0, 5 and 15 min. Data are expressed as the percentage of ERK phosphorylated LSK cells in the LSK cell population. (B) Representative dot plots showing ERK phosphorylated LSK cells when gated on LSK population. (C) LSK cells were sorted out by FACS. After seeding, cells were exposed to LDL 100 µg/ml in the presence or absence of pERK inhibitor, U0126 at 10 µM, for 14 days. Ly-6G^hi^ granulocytes were analyzed by FACS.

As effects of HDL on HSPC are at least in part due to the inhibition of extracellular signal-regulated kinase 1/2 (ERK1/2) [22], we tested if LDL would activate ERK 1/2. After stimulation with 100 µg/ml LDL for 0, 5 or 15 min, total bone marrow cells were fixed and stained with antibodies against lineage markers, Sca-1, c-Kit and pERK. FACS analysis showed that the percentage of pERK positive LSK cells in the LSK population was 9.6±0.53% at baseline level, which increased 2-fold (*P*<0.05) within 5 min after LDL addition and returned to baseline levels after 15 min (Figure 7, A and B). We next exposed LSK cells to LDL with or without the pERK inhibitor, U0126, for 14 days. The myeloid lineage differentiation was analyzed as described above. We found that U0126 abrogated LDL-induced granulocyte differentiation (Figure 7C).

### Pretreatment of LDL enhances Lin− cell motility which is partially mediated via ERK phosphorylation

As the increase of LSK cells in the PB might be due, as suggested by Gomes, to increased mobilization, we tested whether LDL affects adhesion/migration receptor expression and HSPC motility. Lin− cells from wt mice were exposed to LDL for 24 hours and adhesion molecule expression was studied by FACS. Median Fluorescence Intersity (MFI) of CXCR4 was higher in Lin− cells treated with LDL for 24 hours, compared to control (LDL 0 µg/ml: 3857±300.6; LDL 100 µg/ml: 4733±389, n = 9, *P*<0.01). In contrast, LDL treatment did not affect integrin β1 and integrin α5 expression on Lin− cells (integrinβ1: 9874±869.6 vs. 9549±1113.5; integrin α5: 3611±560.2 vs. 3826±496.8, n = 4–5).

**Figure 8:**
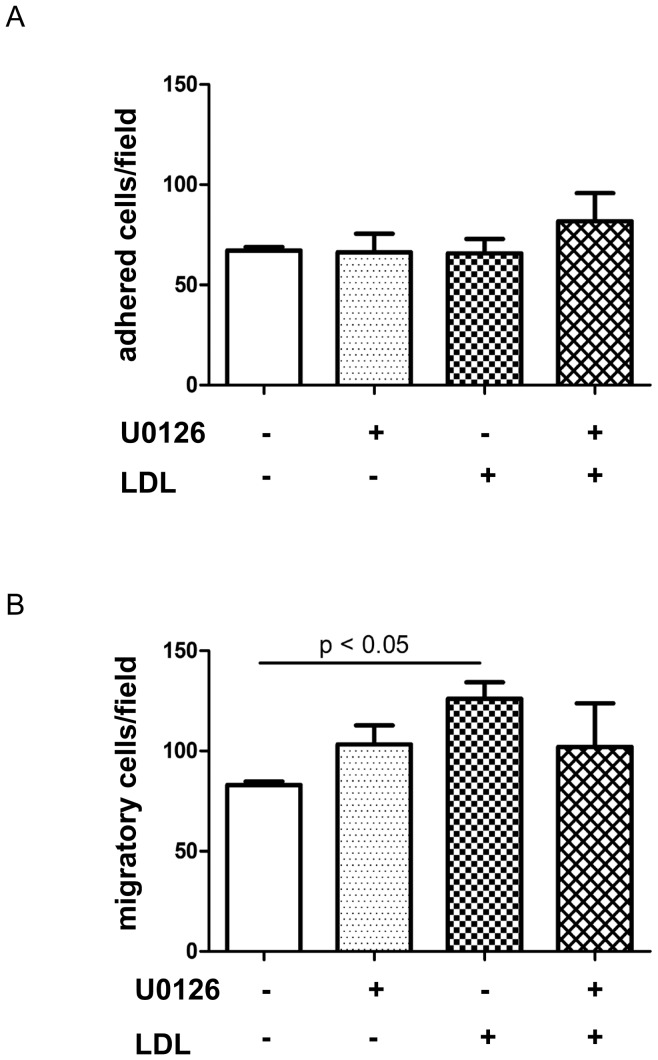
LDL modulates Lin- cell motility which is partially mediated via pERK. To study the effect of LDL on HSPC function, Lin- cells of CD45.2 mice were exposed to 0 or 100 µg/ml LDL in the presence or absence of pERK inhibitor U0126 for 24 hours and then subjected for adhesion and migration assays. (A) Cells were seeded onto fibronectin-coated plates for 4 hours. After extensive washing, adhered cells were stained with crystal violet and counted under the microscopy. (B) Cells were loaded into transwells and allowed to migrate for 4 hours. Cells in the upper chamber of the transwells were removed by swaps. Cells migrated to the lower surface of the transwells were stained with Hoechst. Adherent cells or migrated cells were numerated under the microscope, averaging the number of cells in 5 random fields.

Next, we performed adhesion and migration assays to explore the impact of LDL on Lin− cell mobility. As pERK plays a critical role in the regulation of LDL on HSPC, we also investigated whether modulation of LDL on Lin− cell function required ERK phosphorylation. Lin− cells isolated from WT mice were exposed to 0 or 100 µg/ml LDL in the presence or absence of U0126 for 24 hours and then subjected for adhesion and migration assays. LDL and pERK inhibitor did not change Lin− cell adhesion to fibronectin-coated plates (Figure 8A, n = 5–7), but Lin− cells pretreated with LDL showed increased migration to the lower chamber of a modified Boyden chamber, compared to control (Figure 8B, n = 5–7).

## Discussion

Hypercholesterolemia is at least partially associated with monocytosis because of increased monocyte survival and continued cell proliferation [14]. Noteworthy, infusion of rHDL attenuated monocytosis and neutrophilia in apoE^−/−^ mice on western diet [32]. We here asked the question whether the effects seen on leukocytosis and monocytosis could at least in part be caused by effects of LDL and HDL on HSPC and progenitors.

We describe that LDL and HDL have opposing effects on HSPC behavior. (1) Hypercholesterolemia was associated with leukocytosis, and in particular with increased levels of Ly-6c^hi^ and F4/80^+^ monocytes and Ly-G^hi^ granulocyte production in blood. (2) LDL promoted HSPC differentiation toward atherogenic monocytes and granulocytes *in vitro*, which was inhibited by HDL. (3) LDL stimulated ERK phosphorylation in LSK cells and LDL-promoted LSK differentiation toward granulocytes was partially dependent on ERK phosphorylation. (4) LDL also induces an increase in LSK cells in BM and PB, at least in part due to increased proliferation of HSPC. (5) As LDL also increases motility of HSPC, it is possible that part of the increased number of LSK cells in PB is due to mobilization in response to LDL, as suggested by Gomez.

Although the white blood cell count and HSPC percentage in blood and BM of LDLr^−/−^ mice on normal diet was similar to that of WT mice, the percentage of Ly-6C^hi^, F4/80^+^ monocytes and Ly-6G^hi^ granulocytes was higher in LDLr^−/−^ mice on normal diet vs. WT mice. When LDLr^−/−^ mice were placed on high fat diet, an increased percentage of HSPC and production of these inflammatory cells was seen, compared to LDLr^−/−^ mice on normal diet. As the lipoprotein profile in the blood of WT and LDLr^−/−^ mice on normal diet only differs in LDL levels, the difference in the percentage of monocytes and granulocytes can only be explained by the elevated LDL levels. Therefore, (1) LDL appears to be a potent stimulator for HSPC differentiation into Ly-6c^hi^ and F4/80^+^ monocytes and Ly-G^hi^ myeloid cells; (2) The effect of LDL on HSPC seems to be dose- dependent: mild hypercholesterolemia, such as in LDLr^−/−^ mice on normal diet, causes an increase in myelomonocytic cells in the blood, while high cholesterol levels cause a much more pronounced effect both on proliferation, mobilization and differentiation of HSPC. Consistently, when isolated LSK cells were treated with LDL for 14 days *in vitro*, skewed differentiation of LSK cells toward myeloid cells was seen. Furthermore, LDL-induced HSPC differentiation toward granulocytes was inhibited by addition of the pERK inhibitor, U0126. These *in vitro* data demonstrate a direct positive regulation of LDL on HSPC differentiation into myeloid cell population. Distinct from LDL, addition of HDL prohibited the effect of LDL on HSPC number and differentiation *in vitro*, suggesting the direct but negative regulation of HDL on HSPC.

The anti-atherogenic properties of HDL include reversed cholesterol transport, anti-inflammation, anti-oxidation, maintenance of endothelial integrity and suppression of HSC proliferation in BM [21], [22], [40], [41]. Consistently, our data demonstrated that rHDL inhibited HSPC proliferation. Our data suggest a direct modulation of HSPC by HDL as ABCA1 and SR-BI, the HDL receptors, are expressed on LSK cells; and infusion of apoA-I, like rHDL, decreased LSK cell frequency and proliferation *in vivo*.

Gomes *et al.*, reported that hypercholesterolemia promotes HSPC mobilization from BM partially via enhanced SDF-1 production and breakdown of the SDF-1/CXCR4 axis. *In vitro*, they showed that LDL induced HSPC differentiation into monocytes and this was inhibited by Blt3, a SR-BI inhibitor. Furthermore, LDL-promoted HSPC migration was mediated via Blt3. These data indicate that SR-BI may play a critical role in HSPC biology. During the final preparation of our manuscript, we noticed that Murphy *et al.*, reported the impact of impaired cholesterol efflux on HSPC proliferation and monocyte production in apoE deficient (apoE^−/−^) mice [32]. Our data in LDLr^−/−^ mice are consistent with their findings. Our studies, however, also identify a direct effect of LDL on HSPC differentiation toward Ly-6c^hi^ and F4/80^+^ monocytes and Ly-6G^hi^ granulocytes which is counteracted by HDL. Moreover, short term treatment of LDL enhanced Lin- cell mobility. Given that stem cell proliferation is in close context with its mobilization activity [30], [42], [43], [44], [45], [46], our data of HSPC proliferation and increased Lin− mobility indicate that LDL may promote HSPC mobilization. Consistent with the observation of Yvan-Charvet *et al.*, that pERK is a key signaling molecule in the regulation of lipoproteins in HSPC, we demonstrate that the regulation of LDL on HSPC differentiation into myeloid cells and on Lin− motility are both mediated at least in part by pERK.

Leukocytosis is a marker of inflammation that is associated with ischemic vascular disease, sickle cell disease, and diabetes [47]. Others and we demonstrate that LDL promotes HSPC proliferation, differentiation and leukocytosis while HDL has the opposite effect [32], [48]. However, whether and how LDL-induced HSPC expansion and the associated leukocytosis/monocytosis affects atherosclerosis is still unknown. Moreover, LSK cells contain various subsets of progenitors that may have differential effects on disease development. Lineage tracing studies will be needed to demonstrate the direct contribution of BM LSK cells or other cell populations to atherosclerosis.

Based on our findings and published data, we propose a model defining the influence of lipoproteins on HSPC. LDL promotes HSPC proliferation, leading to increased production of HSPC and GMP, in the blood, as well as myelomonocytic differentiation leading to increased frequency of monocytes and neutrophils in BM and blood. The effects of LDL appear to be counteracted by HDL.

## Supporting Information

Figure S1 **Hypercholesterolemia increased the percentage of proatherogenic monocytes and granulocytes in peripheral blood of LDLr^–/–^ mice.** After red blood cells were lysed, white blood cells were stained with antibodies against CD11b, Ly-6c, F4/80 and Ly-6G for FACS analysis. (A) Ly-6c^hi^ monocytes were indicated in the box. (B) F4/80^+^ monocytes were shown in the box. (C) Ly-6G^hi^ granulocytes were shown in the box. n = 5–7. The percentage of Ly-6c^hi^ and F4/80^+^ monocytes and Ly-6G^hi^ granulocytes was shown on the right corner of each plot.
(TIF)

Figure S2 **Different effects of LDL and rHDL on HSPC** ***in vivo*** **.** (A) Representative FACS plots showing LSK cells in LDLr^−/−^ mice on normal and high fat diet. (B) WT mice received saline, PLPC or rHDL at 4, 8, 12 and 16 mg/kg. Representative FACS plots of LSK cells following saline, PLPC and rHDL treatment. Both A and B were gated on Lin- cells. The percentage of LSK cells in TBMC is indicated on the right corner of each plot.
(TIF)

Figure S3 **LDL induced HSPC differentiation toward myeloid lineage** ***in vitro*** **.** LSK cells sorted by FACS were cultured in LDL or LDL plus HDL for 14 days. LSK cells were cultured in serum free medium with SCF, IL-3, IL-6 and either GM-CSF, LDL, or a combination of LDL and HDL. Cells were harvested and followed by cytospin and Giemsa stains. Representative pictures of cell morphology identified by Giemsa stain: control (A); GM-CSF 10 ng/ml (B); LDL 100 µg/ml (C); and LDL plus HDL (600 µg/ml) (D). Scale bar: 20 µm. n = 3–4 from pooled mice. Red arrow indicates inactive cells; Black arrow indicates promonocytes; Blue arrow indicates granulocytes.
(TIF)

## References

[1] Baigent C, Keech A, Kearney PM, Blackwell L, Buck G, et al (2005) Efficacy and safety of cholesterol-lowering treatment: prospective meta-analysis of data from 90,056 participants in 14 randomised trials of statins. Lancet 366: 1267–1278. 16214597 10.1016/S0140-6736(05)67394-1

[2] Gordon DJ, Probstfield JL, Garrison RJ, Neaton JD, Castelli WP, et al (1989) High-density lipoprotein cholesterol and cardiovascular disease. Four prospective American studies. Circulation 79: 8–15. 2642759 10.1161/01.cir.79.1.8

[3] Refolo LM, Malester B, LaFrancois J, Bryant-Thomas T, Wang R, et al (2000) Hypercholesterolemia accelerates the Alzheimer's amyloid pathology in a transgenic mouse model. Neurobiol Dis 7: 321–331. 10964604 10.1006/nbdi.2000.0304

[4] Skalen K, Gustafsson M, Rydberg EK, Hulten LM, Wiklund O, et al (2002) Subendothelial retention of atherogenic lipoproteins in early atherosclerosis. Nature 417: 750–754. 12066187 10.1038/nature00804

[5] Weber C, Zernecke A, Libby P (2008) The multifaceted contributions of leukocyte subsets to atherosclerosis: lessons from mouse models. Nat Rev Immunol 8: 802–815. 18825131 10.1038/nri2415

[6] Anzinger JJ, Chang J, Xu Q, Buono C, Li Y, et al (2010) Native low-density lipoprotein uptake by macrophage colony-stimulating factor-differentiated human macrophages is mediated by macropinocytosis and micropinocytosis. Arterioscler Thromb Vasc Biol 30: 2022–2031. 20634472 10.1161/ATVBAHA.110.210849 PMC3170564

[7] Rajavashisth TB, Andalibi A, Territo MC, Berliner JA, Navab M, et al (1990) Induction of endothelial cell expression of granulocyte and macrophage colony-stimulating factors by modified low-density lipoproteins. Nature 344: 254–257. 1690354 10.1038/344254a0

[8] Hohensinner PJ, Kaun C, Rychli K, Niessner A, Pfaffenberger S, et al (2007) Macrophage colony stimulating factor expression in human cardiac cells is upregulated by tumor necrosis factor-alpha via an NF-kappaB dependent mechanism. J Thromb Haemost 5: 2520–2528. 17922812 10.1111/j.1538-7836.2007.02784.x

[9] Zhu SN, Chen M, Jongstra-Bilen J, Cybulsky MI (2009) GM-CSF regulates intimal cell proliferation in nascent atherosclerotic lesions. J Exp Med 206: 2141–2149. 19752185 10.1084/jem.20090866 PMC2757868

[10] Saitoh T, Kishida H, Tsukada Y, Fukuma Y, Sano J, et al (2000) Clinical significance of increased plasma concentration of macrophage colony-stimulating factor in patients with angina pectoris. J Am Coll Cardiol 35: 655–665. 10716468 10.1016/s0735-1097(99)00583-5

[11] Michowitz Y, Goldstein E, Roth A, Afek A, Abashidze A, et al (2005) The involvement of tumor necrosis factor-related apoptosis-inducing ligand (TRAIL) in atherosclerosis. J Am Coll Cardiol 45: 1018–1024. 15808757 10.1016/j.jacc.2004.12.065

[12] Goossens P, Gijbels MJ, Zernecke A, Eijgelaar W, Vergouwe MN, et al (2010) Myeloid type I interferon signaling promotes atherosclerosis by stimulating macrophage recruitment to lesions. Cell Metab 12: 142–153. 20674859 10.1016/j.cmet.2010.06.008

[13] Sugiyama S, Okada Y, Sukhova GK, Virmani R, Heinecke JW, et al (2001) Macrophage myeloperoxidase regulation by granulocyte macrophage colony-stimulating factor in human atherosclerosis and implications in acute coronary syndromes. Am J Pathol 158: 879–891. 11238037 10.1016/S0002-9440(10)64036-9 PMC1850342

[14] Swirski FK, Libby P, Aikawa E, Alcaide P, Luscinskas FW, et al (2007) Ly-6Chi monocytes dominate hypercholesterolemia-associated monocytosis and give rise to macrophages in atheromata. J Clin Invest 117: 195–205. 17200719 10.1172/JCI29950 PMC1716211

[15] Gordon DJ, Rifkind BM (1989) High-density lipoprotein–the clinical implications of recent studies. N Engl J Med 321: 1311–1316. 2677733 10.1056/NEJM198911093211907

[16] Khera AV, Cuchel M, de la Llera-Moya M, Rodrigues A, Burke MF, et al (2011) Cholesterol efflux capacity, high-density lipoprotein function, and atherosclerosis. N Engl J Med 364: 127–135. 21226578 10.1056/NEJMoa1001689 PMC3030449

[17] Yvan-Charvet L, Kling J, Pagler T, Li H, Hubbard B, et al (2010) Cholesterol efflux potential and antiinflammatory properties of high-density lipoprotein after treatment with niacin or anacetrapib. Arterioscler Thromb Vasc Biol 30: 1430–1438. 20448206 10.1161/ATVBAHA.110.207142 PMC2917780

[18] Patel S, Di Bartolo BA, Nakhla S, Heather AK, Mitchell TW, et al (2010) Anti-inflammatory effects of apolipoprotein A-I in the rabbit. Atherosclerosis 212: 392–397. 20609437 10.1016/j.atherosclerosis.2010.05.035

[19] Feng Y, van Eck M, Van Craeyveld E, Jacobs F, Carlier V, et al (2009) Critical role of scavenger receptor-BI-expressing bone marrow-derived endothelial progenitor cells in the attenuation of allograft vasculopathy after human apo A-I transfer. Blood 113: 755–764. 18824596 10.1182/blood-2008-06-161794

[20] Feng Y, Gordts SC, Chen F, Hu Y, Van Craeyveld E, et al (2011) Topical HDL administration reduces vein graft atherosclerosis in apo E deficient mice. Atherosclerosis 214: 271–278. 20943224 10.1016/j.atherosclerosis.2010.09.024

[21] Feng Y, Jacobs F, Van Craeyveld E, Brunaud C, Snoeys J, et al (2008) Human ApoA-I transfer attenuates transplant arteriosclerosis via enhanced incorporation of bone marrow-derived endothelial progenitor cells. Arterioscler Thromb Vasc Biol 28: 278–283. 18063807 10.1161/ATVBAHA.107.158741

[22] Yvan-Charvet L, Pagler T, Gautier EL, Avagyan S, Siry RL, et al (2010) ATP-binding cassette transporters and HDL suppress hematopoietic stem cell proliferation. Science 328: 1689–1693. 20488992 10.1126/science.1189731 PMC3032591

[23] Gomes AL, Carvalho T, Serpa J, Torre C, Dias S (2010) Hypercholesterolemia promotes bone marrow cell mobilization by perturbing the SDF-1:CXCR4 axis. Blood 115: 3886–3894. 20009035 10.1182/blood-2009-08-240580

[24] Crysandt M, Hilgers RD, Hobe SV, Eisert A, Jost E, et al (2011) Hypercholesterolemia and its association with enhanced stem cell mobilization and harvest after high-dose cyclophosphamide+G-CSF. Bone Marrow Transplant 46: 1426–1429. 21217788 10.1038/bmt.2010.327

[25] Chapman MJ, Goldstein S, Lagrange D, Laplaud PM (1981) A density gradient ultracentrifugal procedure for the isolation of the major lipoprotein classes from human serum. J Lipid Res 22: 339–358. 6787159

[26] Feng Y, Van Craeyveld E, Jacobs F, Lievens J, Snoeys J, et al (2009) Wild-type apo A-I and apo A-I(Milano) gene transfer reduce native and transplant arteriosclerosis to a similar extent. J Mol Med 87: 287–297. 19066833 10.1007/s00109-008-0427-y

[27] Naveiras O, Nardi V, Wenzel PL, Hauschka PV, Fahey F, et al (2009) Bone-marrow adipocytes as negative regulators of the haematopoietic microenvironment. Nature 460: 259–263. 19516257 10.1038/nature08099 PMC2831539

[28] Baldridge MT, King KY, Boles NC, Weksberg DC, Goodell MA (2009) Quiescent haematopoietic stem cells are activated by IFN-gamma in response to chronic infection. Nature 465: 793–797. 10.1038/nature09135 PMC2935898 20535209

[29] Lee SL, Wang Y, Milbrandt J (1996) Unimpaired macrophage differentiation and activation in mice lacking the zinc finger transplantation factor NGFI-A (EGR1). Mol Cell Biol 16: 4566–4572. 8754857 10.1128/mcb.16.8.4566 PMC231455

[30] Hoggatt J, Singh P, Sampath J, Pelus LM (2009) Prostaglandin E2 enhances hematopoietic stem cell homing, survival, and proliferation. Blood 113: 5444–5455. 19324903 10.1182/blood-2009-01-201335 PMC2689046

[31] Tothova Z, Kollipara R, Huntly BJ, Lee BH, Castrillon DH, et al (2007) FoxOs are critical mediators of hematopoietic stem cell resistance to physiologic oxidative stress. Cell 128: 325–339. 17254970 10.1016/j.cell.2007.01.003

[32] Murphy AJ, Akhtari M, Tolani S, Pagler T, Bijl N, et al (2011) ApoE regulates hematopoietic stem cell proliferation, monocytosis, and monocyte accumulation in atherosclerotic lesions in mice. J Clin Invest 121: 4138–4149. 21968112 10.1172/JCI57559 PMC3195472

[33] Gan B, Hu J, Jiang S, Liu Y, Sahin E, et al (2010) Lkb1 regulates quiescence and metabolic homeostasis of haematopoietic stem cells. Nature 468: 701–704. 21124456 10.1038/nature09595 PMC3058342

[34] Viatour P, Somervaille TC, Venkatasubrahmanyam S, Kogan S, McLaughlin ME, et al (2008) Hematopoietic stem cell quiescence is maintained by compound contributions of the retinoblastoma gene family. Cell Stem Cell 3: 416–428. 18940733 10.1016/j.stem.2008.07.009 PMC2646421

[35] Nakada D, Saunders TL, Morrison SJ (2010) Lkb1 regulates cell cycle and energy metabolism in haematopoietic stem cells. Nature 468: 653–658. 21124450 10.1038/nature09571 PMC3059717

[36] Reimers GJ, Jackson CL, Rickards J, Chan PY, Cohn JS, et al (2011) Inhibition of rupture of established atherosclerotic plaques by treatment with apolipoprotein A-I. Cardiovasc Res 91: 37–44. 21354994 10.1093/cvr/cvr057

[37] Cho KH, Kim JR (2009) A reconstituted HDL containing V156K or R173C apoA-I exhibited anti-inflammatory activity in apo-E deficient mice and showed resistance to myeloperoxidase-mediated oxidation. Exp Mol Med 41: 417–428. 19322022 10.3858/emm.2009.41.6.047 PMC2705862

[38] Murphy AJ, Woollard KJ, Suhartoyo A, Stirzaker RA, Shaw J, et al (2011) Neutrophil activation is attenuated by high-density lipoprotein and apolipoprotein A-I in in vitro and in vivo models of inflammation. Arterioscler Thromb Vasc Biol 31: 1333–1341. 21474825 10.1161/ATVBAHA.111.226258

[39] Acton S, Rigotti A, Landschulz KT, Xu S, Hobbs HH, et al (1996) Identification of scavenger receptor SR-BI as a high density lipoprotein receptor. Science 271: 518–520. 8560269 10.1126/science.271.5248.518

[40] Tabet F, Lambert G, Cuesta Torres LF, Hou L, Sotirchos I, et al (2011) Lipid-free apolipoprotein A-I and discoidal reconstituted high-density lipoproteins differentially inhibit glucose-induced oxidative stress in human macrophages. Arterioscler Thromb Vasc Biol 31: 1192–1200. 21330603 10.1161/ATVBAHA.110.222000

[41] Barter PJ, Puranik R, Rye KA (2007) New insights into the role of HDL as an anti-inflammatory agent in the prevention of cardiovascular disease. Curr Cardiol Rep 9: 493–498. 17999875 10.1007/BF02938394

[42] Neben S, Marcus K, Mauch P (1993) Mobilization of hematopoietic stem and progenitor cell subpopulations from the marrow to the blood of mice following cyclophosphamide and/or granulocyte colony-stimulating factor. Blood 81: 1960–1967. 7681707

[43] Morrison SJ, Wright DE, Weissman IL (1997) Cyclophosphamide/granulocyte colony-stimulating factor induces hematopoietic stem cells to proliferate prior to mobilization. Proc Natl Acad Sci U S A 94: 1908–1913. 9050878 10.1073/pnas.94.5.1908 PMC20016

[44] Singh P, Hu P, Hoggatt J, Moh A, Pelus LM (2012) Expansion of bone marrow neutrophils following G-CSF administration in mice results in osteolineage cell apoptosis and mobilization of hematopoietic stem and progenitor cells. Leukemia doi:10.1038/leu.2012.117. 10.1038/leu.2012.117 PMC3410045 22543963

[45] Carlo-Stella C, Di Nicola M, Milani R, Guidetti A, Magni M, et al (2004) Use of recombinant human growth hormone (rhGH) plus recombinant human granulocyte colony-stimulating factor (rhG-CSF) for the mobilization and collection of CD34+ cells in poor mobilizers. Blood 103: 3287–3295. 14726397 10.1182/blood-2003-07-2428

[46] Wright DE, Cheshier SH, Wagers AJ, Randall TD, Christensen JL, et al (2001) Cyclophosphamide/granulocyte colony-stimulating factor causes selective mobilization of bone marrow hematopoietic stem cells into the blood after M phase of the cell cycle. Blood 97: 2278–2285. 11290588 10.1182/blood.v97.8.2278

[47] Coller BS (2005) Leukocytosis and ischemic vascular disease morbidity and mortality: is it time to intervene? Arterioscler Thromb Vasc Biol 25: 658–670. 15662026 10.1161/01.ATV.0000156877.94472.a5

[48] Strawn WB, Ferrario CM (2008) Angiotensin II AT1 receptor blockade normalizes CD11b+ monocyte production in bone marrow of hypercholesterolemic monkeys. Atherosclerosis 196: 624–632. 17692319 10.1016/j.atherosclerosis.2007.06.024 PMC2265080

