# Supplementary figures and images for: Hematopoietic Stem/Progenitor Cell Proliferation and Differentiation Is Differentially Regulated by High-Density and Low-Density Lipoproteins in Mice
Source: PLoS One. 2012 Nov 7;7(11):e47286. doi: 10.1371/journal.pone.0047286 (PMC3492382; doi:10.1371/journal.pone.0047286)

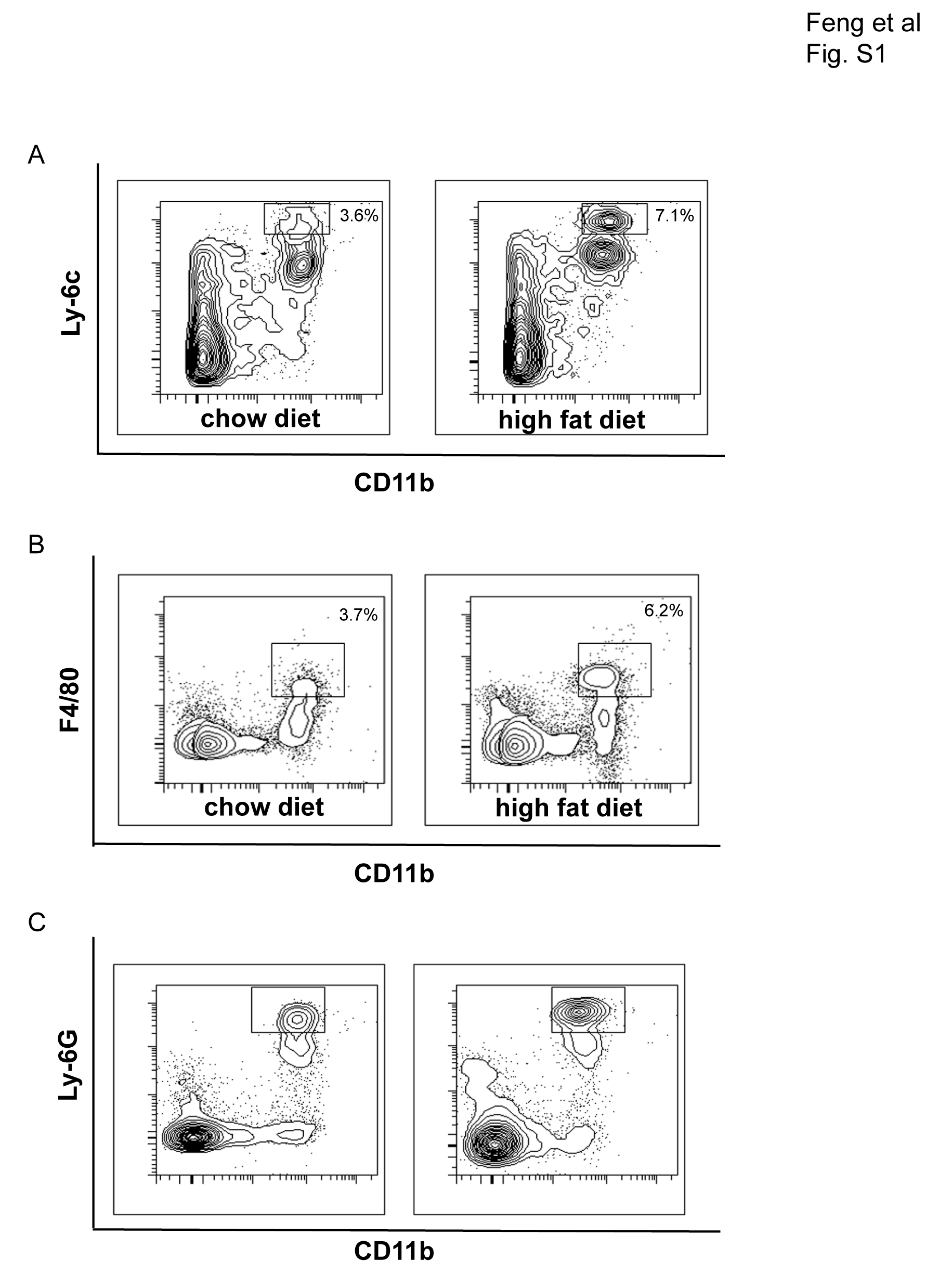

Supplement: Figure S1 — Hypercholesterolemia increased the percentage of proatherogenic monocytes and granulocytes in peripheral blood of LDLr–/– mice. After red blood cells were lysed, white blood cells were stained with antibodies against CD11b, Ly-6c, F4/80 and Ly-6G for FACS analysis. (A) Ly-6chi monocytes were indicated in the box. (B) F4/80+ monocytes were shown in the box. (C) Ly-6Ghi granulocytes were shown in the box. n = 5–7. The percentage of Ly-6chi and F4/80+ monocytes and Ly-6Ghi granulocytes was shown on the right corner of each plot. (TIF) [file pone.0047286.s001.tif]

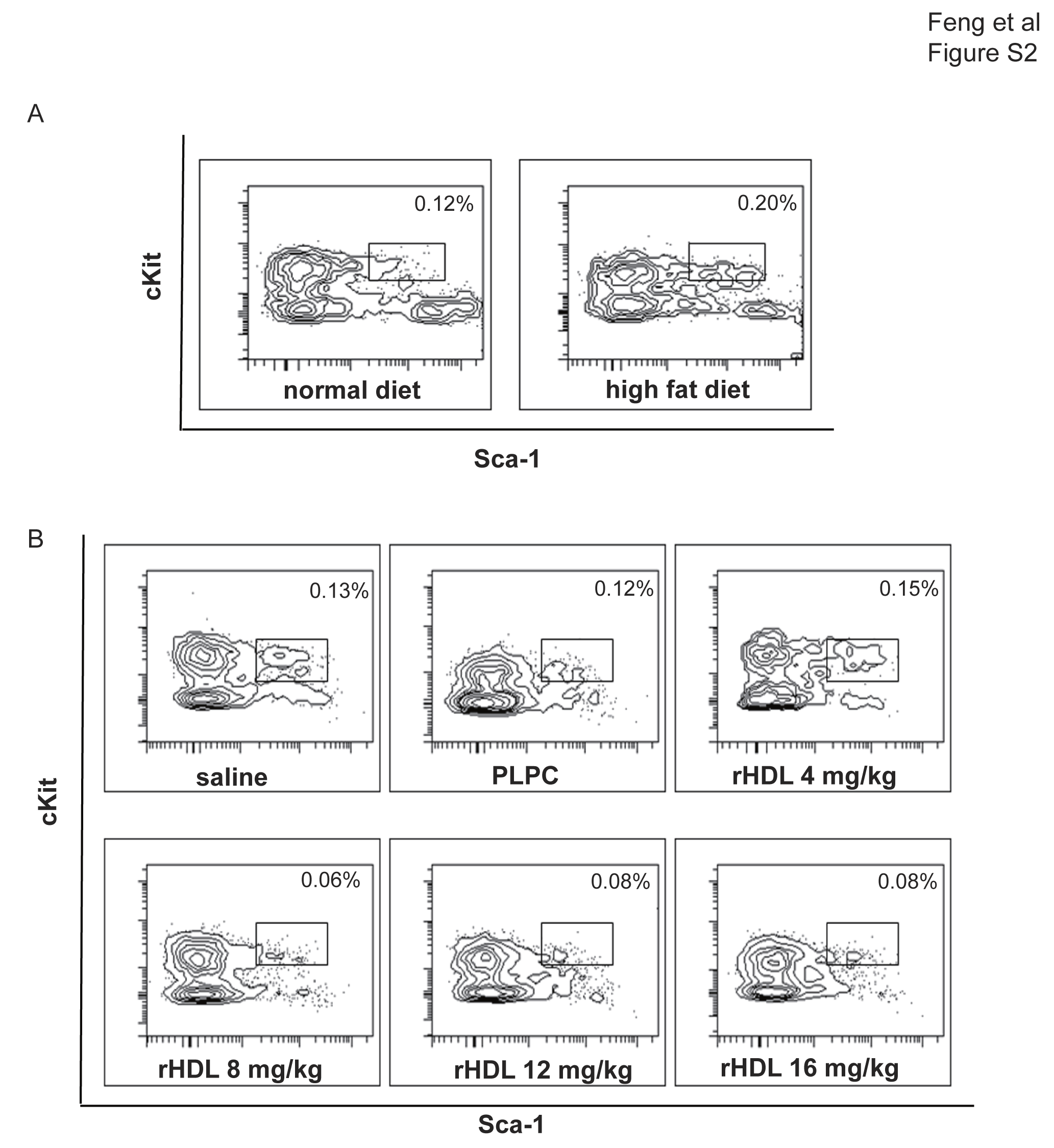

Supplement: Figure S2 — Different effects of LDL and rHDL on HSPC in vivo . (A) Representative FACS plots showing LSK cells in LDLr−/− mice on normal and high fat diet. (B) WT mice received saline, PLPC or rHDL at 4, 8, 12 and 16 mg/kg. Representative FACS plots of LSK cells following saline, PLPC and rHDL treatment. Both A and B were gated on Lin- cells. The percentage of LSK cells in TBMC is indicated on the right corner of each plot. (TIF) [file pone.0047286.s002.tif]

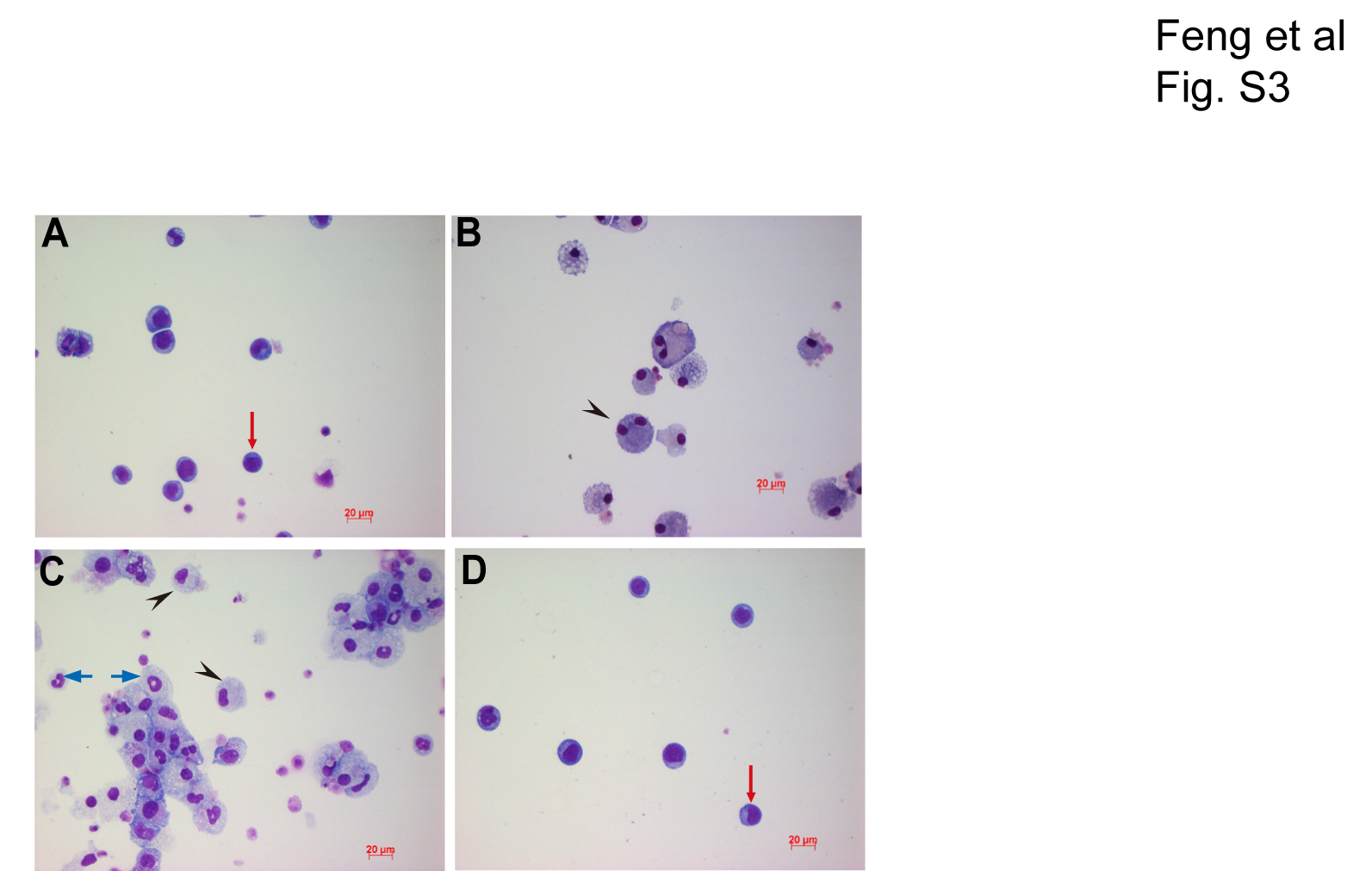

Supplement: Figure S3 — LDL induced HSPC differentiation toward myeloid lineage in vitro . LSK cells sorted by FACS were cultured in LDL or LDL plus HDL for 14 days. LSK cells were cultured in serum free medium with SCF, IL-3, IL-6 and either GM-CSF, LDL, or a combination of LDL and HDL. Cells were harvested and followed by cytospin and Giemsa stains. Representative pictures of cell morphology identified by Giemsa stain: control (A); GM-CSF 10 ng/ml (B); LDL 100 µg/ml (C); and LDL plus HDL (600 µg/ml) (D). Scale bar: 20 µm. n = 3–4 from pooled mice. Red arrow indicates inactive cells; Black arrow indicates promonocytes; Blue arrow indicates granulocytes. (TIF) [file pone.0047286.s003.tif]
